# Associations between single nucleotide polymorphisms of cytokines and hepatitis B virus‐related liver cirrhosis: A case‐control study

**DOI:** 10.1002/iid3.70017

**Published:** 2024-09-24

**Authors:** Yijun Li, Haowei Zhou, Weikang Wu, Wenhua Zhang, Yancheng Ye, Wenling Jia, Chunhui Liang, Haitao Tang, Fengmei Wang, Zhongjun Shao, Xiaojie Yuan, Weilu Zhang

**Affiliations:** ^1^ Department of Epidemiology, Ministry of Education Key Lab of Hazard Assessment and Control in Special Operational Environment, School of Public Health Fourth Military Medical University Xi'an China; ^2^ Education Department of Surgery, Frist Affiliated Hospital Fourth Military Medical University Xi'an China; ^3^ Department of Burns and Plastic Surgery, Second Affiliated Hospital Fourth Military Medical University Xi'an China; ^4^ Clinical Drug Experiment Institution Gansu Wuwei Tumor Hospital Wuwei China; ^5^ Hepatobiliary Center Gansu Wuwei Tumor Hospital Wuwei China

**Keywords:** hepatitis B virus, liver cirrhosis, single‐nucleotide polymorphism

## Abstract

**Background and Aims:**

Various inflammatory and immune cytokines play key roles in the progression of hepatitis B virus (HBV)‐related liver cirrhosis (LC). This study explored the relationship between single nucleotide polymorphisms (SNPs) in cytokines with the combined effect of polymorphisms and gender‐polymorphisms interaction and LC risk.

**Methods:**

In this study, a case–control design was used, samples were selected from 45 patients with hepatitis B‐related cirrhosis and 45 age‐gender‐matched chronic HBV‐infected patients without cirrhosis attending the tumor hospital of Wuwei Academy of Medical Sciences. Fifteen SNPs were examined using a real‐time polymerase chain reaction allelic discrimination system. Logistic regression was utilized to assess cytokine‐associated SNPs and the association between SNPs and LC progression in HBV‐infected patients.

**Results:**

The multivariate‐adjusted logistic model revealed that the GG/AG dominant model (OR, 16.38; 95% CI, 1.13–236.70) and G allele (OR, 5.93; 95% CI, 0.98–36.01) of rs1800896 were associated with an increased risk of cirrhosis in CHB patients. Instead, rs2227306 CT presented a reduced cirrhosis risk (OR, 0.22; 95% CI, 0.04–1.38). Rs2055979 AA/AC was negatively associated with the risk of cirrhosis, potentially reversed in males (*p* = 0.021). Rs1799964 CC/CT was positively related to the risk of cirrhosis but reduced the risk of cirrhosis in males (OR, 0.13; 95% CI, 0.022–0.808; *p* = 0.028). Both rs1799964 TT and rs1799724 CT/TT genotype showed a synergistic effect in reducing the risk of cirrhosis with rs1800896 AA (OR, 0.08; 95% CI, 0.01–1.43 and OR, 0.12; 95% CI, 0.01–2.21).

**Conclusion:**

Polymorphisms rs1800896 and rs2227306 are potentially associated with the risk of cirrhosis. For the first time, the study highlights that the rs2055979 AA/AC and rs1799964 CC/CT polymorphism interact with gender and its potential reversal of cirrhosis risk in males. Furthermore, rs1800896 AA showed a synergistic effect with rs1799964 TT and rs1799724 CT/TT to prevent the progression of HBV infection to cirrhosis.

## 1 INTRODUCTION

Hepatitis B virus (HBV) infection, a global health concern, impacts approximately 316 million individuals worldwide.^1^ China, as a typical developing country, is still facing a significant burden with an HBV prevalence of about 3.0% (95% CI, 2.1%–3.9%) or roughly 43.3 million people.^2^ According to the World Health Organization definition, a prevalence between 5.0% and 7.9% is classified as a higher intermediate prevalence. Wuwei City, located in northwest mainland China, has been selected as a pilot site for HBV prevention and treatment programs due to its higher prevalence of HBV infection. With various prevention and treatment measures, the hepatitis B surface antigen‐positive rate in the area decreased from 7.2% (95% CI, 6.3%–8.1%) reported in 2010 to 4.1% in 2020.^3^, ^4^ This figure remains higher than the national average, and patients with chronic hepatitis B (CHB) are at significantly higher risk for developing liver cirrhosis (LC), which can further lead to hepatocellular carcinoma (HCC) and liver disease‐related mortality. Therefore, impeding CHB progression to LC is crucial for the patient's long‐term health.

Cytokines, which act as critical mediators, regulate inflammatory immune responses and affect the outcome of the HBV infection.^5^ Single nucleotide polymorphisms (SNPs), as host factors, can influence cytokine expression. Cytokine‐associated SNPs have also been linked to the clinical outcomes of HBV infection. The IL‐1β TT genotype has been demonstrated to elevate the likelihood of HBV infection.^6^ The IL‐4 CC genotype and the tumor necrosis factor (TNF‐α) TT genotype have been demonstrated to confer a decreased risk of HBV infection.^7^, ^8^ Both the IL‐12β CC genotype and the transforming growth factor (TGF‐β1) CC genotype have been identified to increase the risk of HCC infection.^9^, ^10^ HBV‐related LC, as an important phase of HBV infection, was found to be related to several SNPs of cytokines, such as the TT genotype of interleukin‐2 (IL‐2),^7^ the TT genotype of IL‐6,^11^ and the CC genotype of IL‐10.^12^ These SNPs have been demonstrated to increase the risk of HBV‐related LC. However, the majority of studies have been independent discoveries of cytokine‐associated SNPs, and the combined effects of multiple SNPs and their association with HBV‐related LC are rare.

In addition to cytokines, cellular receptor polymorphisms have been identified as a factor in HBV infection. Sodium Taurocholate Cotransporting Polypeptide (NTCP), a sodium‐dependent bile acid transport protein that facilitates the movement of bile acids from the blood into hepatocytes, has been identified as a cellular receptor for HBV in host hepatocytes. It has been demonstrated that the rs2296651 GG genotype of NTCP is associated with an increased risk of LC.^13^ Furthermore, the rs4646287 AA genotype has been identified to increase the risk of HCC infection.^14^ Nevertheless, these findings require further cross‐validation by additional studies, and there are still other cytokines to be explored in the context of HBV‐related LC.

Therefore, this study aimed to investigate the associations between SNPs located in cytokine and NTCP genes and HBV‐related LC, particularly focusing on examining the combined effects of these polymorphisms and their interaction with gender.

## 2 MATERIALS AND METHODS

### 2.1 Participants

This was a case–control study. A total of 357 participants with CHB, who had previously tested positive for HBsAg for at least 6 months, were continuously enrolled from Gansu Wuwei Medical Academy Cancer Hospital between August 2018 and January 2021. Among them, 45 participants had previously been diagnosed with cirrhosis (the LC group). After excluding nine participants (seven anti‐HCV positive and two with persistently negative SNP results), and in a 1:1 ratio, participants without HCC or hepatic failure of the same sex and with an age difference of no more than 5 years during the same period were randomly selected as the CHB group. A flow chart of the selection and assignment process is presented in Figure S1. Cirrhosis diagnosis followed the criteria proposed in 2019,^15^ which required the exclusion of noncirrhotic portal hypertension and the presence of at least two of the following five criteria: (1) signs of cirrhosis and/or portal hypertension by imaging; (2) esophageal and gastric varices by endoscopic examination; (3) liver cirrhosis by liver stiffness measure (LSM) (>17.5 kPa); (4) decreased albumin level (<35 g/L) and/or prolonged PT (>3 s compared with the control); (5) platelet count <100 × 10^9^/L.

All research was approved by The Clinical Trial Ethics Committee of Gansu Wuwei Medical Academy Cancer Hospital (2024‐Ethics Approval‐10). All participants were fully informed about the study details, and written informed consent was obtained from the participants themselves or their legal guardians before their participation.

### 2.2 Data collection

Data was collected through structured questionnaire‐based, face‐to‐face interviews by trained investigators. Physical and serological examinations were performed at Gansu Wuwei Medical Academy Cancer Hospital. Liver function parameters were evaluated using standard laboratory tests, such as aspartate aminotransferase (AST), alanine aminotransferase (ALT), total bilirubin (TBIL), direct bilirubin (DBIL), indirect bilirubin (IBIL), gamma‐glutamyl transpeptidase (GGT), and albumin (ALB) concentrations. Serological markers (including HBsAg, HBeAg, anti‐HBs, anti‐HBe, anti‐HBc, and anti‐hepatitis C virus) were tested using enzyme‐linked immunosorbent assay (ELISA) reagents (KHB). Serum HBV DNA levels were measured using a hybrid capture assay (DAAN gene) until 2019, thereafter using a PCR‐fluorescence probing assay (ABBOTT). LSM and controlled attenuation parameter (CAP) were obtained using the Fibroscan® (Echosens) system, which utilizes a one‐dimensional transient elastography technique.

### 2.3 Quantitative examination of pgRNA and HBcrAg

HBV pgRNA was detected by the RNA simultaneous amplification testing method based on real‐time fluorescence detection of RNA transcription‐mediated nucleic acid amplification (Shengxiang Biotechnology).^16^ The assay has a linear measurement range from 2 to 9 log_10_ copies/mL, with levels below 2 log_10_ copies/mL considered negative for HBV pgRNA.

Serum HBcrAg was analyzed using the Lumipulse G HBcrAg chemiluminescence Enzyme Immunoassay (Fujirebio). This immunoassay measures the antigenic reactivity of three proteins: HBeAg, HBcAg, and core‐related protein p22cr (products of the HBV pre‐core/core gene sharing a 149‐amino‐acid sequence). The assay's linear measurement range spans from 3 to 7 log_10_ U/mL. Despite the machine's lowest sensitivity limit being 2 log_10_ U/mL, complete specificity was reached for values above 3 log_10_ U/mL, thus HBcrAg levels between 2 and 3 log_10_ U/mL were considered negative. Samples presenting HBcrAg above 7 log_10_ U/mL were diluted and retested with a specific reagent to quantify HBcrAg values.

### 2.4 Human SNPs

Genomic DNA was isolated from whole blood samples using Human Genome Whole Blood Extraction Kit (Tianlong). Thirteen SNPs related to cytokines (rs2055979 of IL‐21, rs3806798 of IL‐15, rs2069762 of IL‐2, rs1800795 of IL‐6, rs2227306 of CXCL8/IL‐8, rs1800469 of TGF‐β1, rs1800896 of IL‐10/IL‐19, rs1061624 of TNFRSF1B, rs1799724 of TNF‐α, rs12979860 of IFNL4/IL‐28B, rs1799964 of TNF‐α, rs1143634 of IL‐1β, rs1800872 of IL‐10/IL‐19) were assessed with TaqMan® MGB SNP Genotyping Kit (Fuyuan Biotechnology Co. Ltd) using a real‐time polymerase chain reaction (PCR) allelic discrimination system (Tianlong). The universal reaction conditions for cytokine‐related SNP genotyping were as follows: 5 μL DNA, 2 μL primer & probe premix, and 10 μL RealFAST Probe PCR mix made to a final volume of 25 μL with 8 μL double distilled water. The PCR cycle conditions consisted of an initial denaturation at 95°C for 5 min followed by 40 cycles at 95°C for 10 s and 60°C for 30 s. Primers for 13 SNPs were listed in Table S1. Shannon entropy (SE) of human SNPs was calculated according to the following formula,^17^ where i represents the genotype and pi represents the percentage of each genotype.

$$\mathrm{SE}=-\sum_{i}^{n}p_{i}\times \ln\;\left(p_{i}\right).$$

In addition, we assessed two SNPs related to sodium taurocholate co‐transporting polypeptide (NTCP) (rs2296651 and rs4646287 of SLC10A1) by the TaqMan® SNP Genotyping Assay (ABI) according to manufacturer's instruction.

### 2.5 Statistical analysis

Variables were presented as means with corresponding standard deviations (SD), medians with quartiles, or as simple numbers with percentages, and were compared using Student's *T* test, Wilcoxon's test, standard *χ* ^2^ tests, or Fisher's Exact tests. The Hardy–Weinberg equilibrium was determined by the *χ* ^2^ test. In consideration of linkage disequilibrium and the combined effect of SNPs, the linkage disequilibrium (LD) between SNPs was calculated using the SHEsis online software platform (http://analysis.bio-x.cn), with *r* ^2^ as the coefficient and *r* ^2^ > 0.8 indicating a significant linkage disequilibrium between two loci. The associations between SNPs and LC risk were examined using univariate and multivariate logistic regression models, with results expressed as odds ratios (OR) with 95% confidence intervals (CI). A stratified analysis was performed and the interaction effect was examined according to gender. The interaction effect between SNPs was also analyzed. Statistical analyses were performed using SAS version 9.4 (SAS Institute Inc) and with R software, version 4.3.0 (http://cran.r-project.org/). A *p* value of 0.05 was established as the threshold for statistical significance and a *p* value between 0.05 and 0.10 was considered to have potential statistical significance.

## 3 RESULTS

### 3.1 Baseline characteristic

Table 1 summarizes participants' characteristics, including demographic information, biochemical features, and virological data. Forty‐five individuals with CHB with a mean age of 55.4 (7.5) years and 45 individuals with LC with a mean age of 54.4 (8.1) years were enrolled in this study, which was not significantly different. The LC group had higher proportions of smoking, antiviral treatment, and higher levels of HBcrAg, ALT, AST, and LSM. However, the proportions of alcohol drinking and HBeAg(+), and the levels of HBV DNA, HBV RNA, and CAP were significantly lower than the CHB group.

**Table 1:** Clinicopathological characteristics of participants.

Characteristics	CHB (*n* = 45)	LC (*n* = 45)	*p* Value
Age, years	55.4 ± 7.5	54.4 ± 8.1	0.543
Gender, male (%)	30 (66.7)	30 (66.7)	1.000
Smoking, *n* (%)	17 (37.8)	23 (51.1)	0.203
Drinking, *n* (%)	5 (11.1)	2 (4.4)	0.434
Antiviral treatment, *n* (%)	18 (40.0)	43 (95.6)	<0.001
HBeAg+, *n* (%)	16 (35.6)	5 (11.1)	0.006
HBV DNA, log_10_ IU/mL	3.1 (2.0–4.9)	2.0 (2.0–2.0)	<0.001
HBV RNA, log_10_ IU/mL	3.1 (2.0–4.1)	2.0 (2.0–3.0)	0.002
HBcrAg,^a^ log_10_ U/mL	4.0 (3.0–7.2)	4.8 (3.4–5.5)	0.961
ALT, IU/L	40.0 (25.9–71.0)	41.1 (23.8–61.3)	0.608
AST, IU/L	30.0 (23.0–49.8)	37.4 (28.3–49.7)	0.108
CAP, dB/m	235.0 (207.0–275.0)	218.0 (181.0–240.0)	0.015
LSM, kPa	5.3 (3.8–7.6)	14.2 (8.7–18.0)	<0.001

Abbreviations: ALT, alanine aminotransferase; AST, aspartate aminotransferase; CAP, controlled attenuation parameter; CHB, chronic hepatitis B; HBcrAg, hepatitis B c antigen; HBeAg, Hepatitis B e antigen; HBV, hepatitis B virus; LC, liver cirrhosis; LSM, liver stiffness measurement.

^a^ HBcrAg results of six participants were missing, two in CHB, and four in LC group.

**Figure 1:**
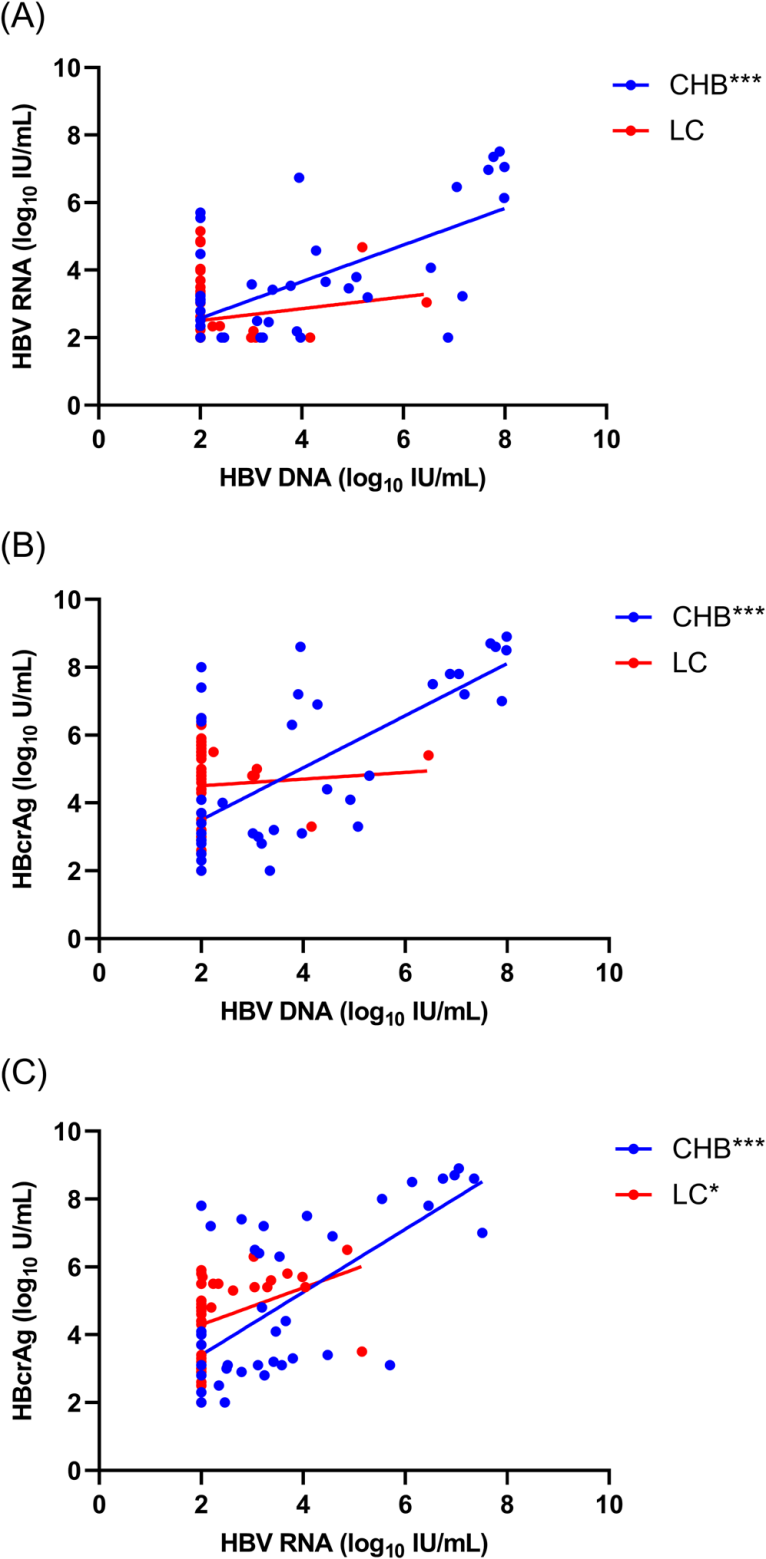
Correlation analysis of HBV DNA, HBV RNA, and HBcrAg between the CHB and LC groups. (A) Association between HBV DNA and HBV RNA in CHB and LC groups. (B) Association between HBV DNA and HBcrAg in CHB and LC groups. (C) Association between HBV RNA and HBcrAg in CHB and LC groups. ***p < 0.001. *p < 0.05. CHB, chronic hepatitis B; LC, liver cirrhosis.

Surprisingly, HBV DNA, HBV RNA, and HBcrAg were highly correlated in CHB (*r* = 0.657, *r* = 0.694 and *r* = 0.683, *p* < 0.001, respectively), but not in LC participants. In the LC group, only a positive correlation between HBV RNA and HBcrAg (*r* = 0.376, *p* = 0.016) was observed (Figure 1). Multivariate logistic regression showed HBV RNA to be more representative of HBV cirrhosis than HBV DNA (Table S2). Moreover, factors such as antiviral treatment, and HBeAg status were also related to HBV‐related LC (Table S2).

### 3.2 Distributions of the SNPs

The genotypic distribution of rs2055979, rs3806798, rs2069762, rs1800795, rs2227306, rs1800469, rs1800896, rs1061624, rs1799724, rs12979860, rs1799964, rs1143634, rs1800872, rs2296651, and rs4646287 polymorphism complied with the Hardye–Weinberg equilibrium (*p* > 0.05, Table S3). We genotyped fifteen polymorphisms in CHB and LC patients and depicted their genotypic distribution in Figure S2. Among them, rs2069762 showed a significant difference between the CHB and LC groups, with the LC group having a lower proportion of GG (*p* = 0.036). Meanwhile, rs1799964 and rs1800469 showed a probable difference between the CHB and LC groups, with the LC group having a higher proportion of CC (*p* = 0.054) and a lower proportion of TT (*p* = 0.086).

### 3.3 SNPs and HBV‐related LC

To evaluate the effect of cytokine‐related SNPs on HBV progression, we constructed univariate and multivariate‐adjusted logistic models for each SNP based on genotype, dominant and recessive model, and allele frequency (Tables 2, S4–S7). After adjusting for age, gender, antiviral treatment, HBeAg status, HBV RNA, HBcrAg, and alcohol drinking, rs1800896 GG/AG presented an increased risk of cirrhosis (OR, 16.38; 95% CI, 1.13–236.70; *p *= 0.040, Table 2). The G allele also showed an increased risk of cirrhosis (OR, 5.93; 95% CI, 0.98–36.01; *p* = 0.053, Table 2).

**Table 2:** Association between cytokines‐related SNPs and cirrhosis in chronic hepatitis B infection participants.

Genotype	CHB (*n* = 45)	LC (*n* = 45)	Univariate model		Multivariate model^a^	
			OR (95% CI)	*p* Value	OR (95% CI)	*p* Value
**rs1800896**						
AA	36 (80.0)	39 (86.7)	1.00		1.00	
AG	8 (17.8)	6 (13.3)	0.69 (0.22–2.19)	0.969	16.37 (1.13–236.40)	0.979
GG	1 (2.2)	0 (0.0)	‐	‐	‐	‐
Dominant						
AA	36 (80.0)	39 (86.7)	1.00		1.00	
GG/AG	9 (20.0)	6 (13.3)	0.62 (0.20–1.90)	0.399	16.38 (1.13–236.70)	0.040
Recessive						
AA/AG	44 (97.8)	45 (100)	1.00		1.00	
GG	1 (2.2)	0 (0.0)	‐	‐	‐	‐
Allele						
A	80 (88.9)	84 (93.3)	1.00		1.00	
G	10 (11.1)	6 (6.7)	0.57 (0.20–1.65)	0.300	5.93 (0.98–36.01)	0.053
**rs2227306**						
CC	19 (42.2)	16 (35.6)	1.00		1.00	
CT	16 (35.6)	19 (42.2)	1.41 (0.55–3.61)	0.558	0.22 (0.04–1.38)	0.043
TT	10 (22.2)	10 (22.2)	1.19 (0.40–3.57)	1.000	2.65 (0.25–27.80)	0.145
Dominant						
CC	19 (42.2)	16 (35.6)	1.00		1.00	
TT/CT	26 (57.8)	29 (64.4)	1.33 (0.57–3.10)	0.517	0.53 (0.12–2.42)	0.410
Recessive						
CC/CT	35 (77.8)	35 (77.8)	1.00		1.00	
TT	10 (22.2)	10 (22.2)	1.00 (0.37–2.70)	1.000	4.46 (0.51–38.96)	0.176
Allele						
C	54 (60.0)	51 (56.7)	1.00		1.0	
T	36 (40.0)	39 (43.3)	1.15 (0.63–2.08)	0.650	1.11 (0.41–2.98)	0.839
**rs2069762**						
TT	13 (28.9)	25 (55.6)	1.00		1.00	
TG	27 (60.0)	17 (37.8)	0.33 (0.13–0.81)	0.293	0.75 (0.16–3.51)	0.752
GG	5 (11.1)	3 (6.7)	0.31 (0.06–1.52)	0.429	0.32 (0.02–4.43)	0.440
Dominant						
TT	13 (28.9)	25 (55.6)	1.00		1.00	
GG/TG	32 (71.1)	20 (44.4)	0.33 (0.14–0.78)	**0.012**	0.66 (0.15–2.86)	0.574
Recessive						
TT/TG	40 (88.9)	42 (93.3)	1.00		1.00	
GG	5 (11.1)	3 (6.7)	0.57 (0.13–2.55)	0.463	0.37 (0.03–4.63)	0.443
Allele						
T	53 (58.9)	67 (74.4)	1.00		1.00	
G	37 (41.1)	23 (25.6)	0.49 (0.26–0.93)	**0.028**	0.68 (0.24–1.95)	0.477

Abbreviations: A, adenine; C, cytosine; CHB, chronic hepatitis B; CI, confidence interval G, guanine; LC, liver cirrhosis; OR, odds ratio; T, thymine.

^a^ Adjusting for age, gender, smoking, alcohol drinking, antiviral treatment, HBeAg status, HBV RNA, and HBcrAg.

In terms of genotype, only rs2227306 CT presented a reduced cirrhosis risk after adjusting for age, gender, antiviral treatment, HBeAg status, HBV RNA, HBcrAg, and alcohol drinking in the multivariate model (OR, 0.22; 95% CI, 0.04–1.38; *p *= 0.043, Table 2). Upon stratifying participants by the dominant model, individuals with the TT/CT genotype of rs2227306 exhibited lower levels of HBV DNA (Table S8).

Similarly, rs2069762 GG/TG as the dominant model (OR, 0.33; 95% CI, 0.14–0.78; *p* = 0.012) and the G allele (OR, 0.49; 95% CI, 0.26–0.93; *p *= 0.028) presented a reduced cirrhosis risk only in the univariate model (Table 2). No associations were observed with two SNPs related to NCTP during the liver cirrhosis progression in patients with chronic HBV infection (Table 2).

### 3.4 Gender difference

The interaction between gender and SNPs was further analyzed. A potential interaction was observed between gender and rs2055979, and rs1799964.

Rs2055979 AA/AC was negatively associated with the risk of cirrhosis, which appeared to reverse for male participants (*p *= 0.021, Table 3). However, when stratifying participants by gender, the multivariate‐adjusted regression analysis did not reveal a significant association between rs2055979 and cirrhosis for either gender (data not shown).

**Table 3:** Interaction analysis of rs2055979 with gender.

Parameter		*β*	SE	Wald *χ* ^2^	*p* Value
Intercept		−3.979	3.923	1.029	0.310
rs2055979	AA/AC	−0.715	0.575	1.543	0.214
Gender	Male	−0.837	0.540	2.406	0.121
rs2055979 × gender	AA/AC	1.606	0.696	5.322	0.021
Age, years		0.024	0.052	0.202	0.653
Antiviral treatment	Yes	3.426	1.060	10.449	0.001
HBeAg, log_10_ IU/mL	+	−1.565	0.590	7.027	0.008
HBV RNA, log_10_ IU/mL		−1.663	0.532	9.753	0.002
HBcrAg, log_10_ U/mL		0.922	0.406	5.146	0.023
Drinking	Yes	−0.609	0.763	0.637	0.425

Abbreviations: C, cytosine; HBcrAg, hepatitis B c antigen; HBeAg, hepatitis B e antigen; HBV, hepatitis B virus; T, thymine.

Instead, rs1799964 CC/CT was positively associated with the risk of cirrhosis, which appeared to reverse for male participants (*p *= 0.093, Table 4). However, when stratifying participants by gender, the multivariate‐adjusted regression analysis showed that rs1799964 CC/CT reduced the risk of cirrhosis in males (OR, 0.13; 95% CI, 0.022–0.808; *p *= 0.028, Table S9).

**Table 4:** Interaction analysis of rs1799964 with gender.

Parameter		*β*	SE	Wald *χ* ^2^	*p* Value
Intercept		−2.895	3.880	0.557	0.456
rs1799964	CC/CT	0.119	0.485	0.061	0.806
Gender	Male	−0.505	0.458	1.219	0.270
rs1799964 × gender	CC/CT	−0.818	0.487	2.819	0.093
Age, years		0.017	0.053	0.102	0.750
Antiviral treatment		2.407	0.637	14.264	<0.001
HBeAg, log_10_ IU/mL		−1.393	0.568	6.004	0.014
HBV RNA, log_10_ IU/mL		−0.711	0.365	3.787	0.052
HBcrAg, log_10_ U/mL		0.414	0.331	1.561	0.212
Drinking	Yes	−0.301	0.903	0.111	0.739

Abbreviations: C, cytosine; HBcrAg, hepatitis B c antigen; HBeAg, hepatitis B e antigen; HBV, hepatitis B virus; T, thymine.

### 3.5 Combination effect of SNPs

**Figure 2:**
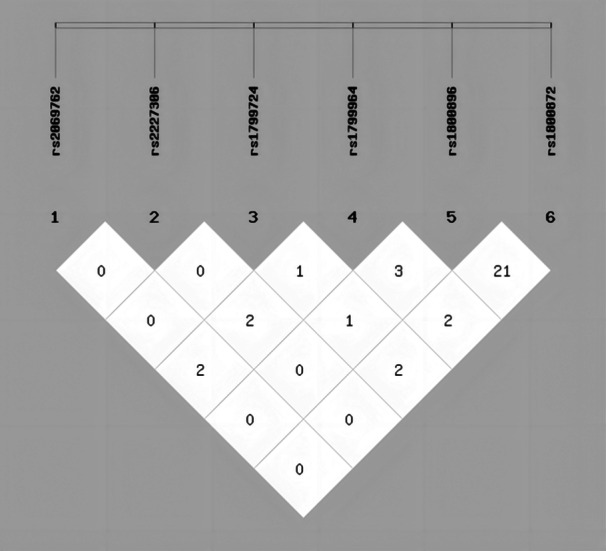
Linkage disequilibrium tests among the rs2069762, rs2227306, rs1799724, rs1799964, rs1800896, and rs1800872 between the CHB and LC groups. CHB, chronic hepatitis B; LC, liver cirrhosis.

To understand the collective effect of gene polymorphisms associated with pro‐ and anti‐inflammatory cytokines on immunoregulation, we investigated the interactions between rs2069762, rs2227306, rs1799724, rs1799964, rs1800896, and rs1800872 in subjects with HBV infections. Pairwise LD analysis between different SNPs was conducted using SHEsis software (Figure 2). No significant LD was found among any pair of gene polymorphisms in our study population, indicating the SNPs were independent (*r* ^2^ < 0.5, Table S10). A potential combined effect to reduce the risk of cirrhosis was found with the rs1800896 AA genotype and the rs1799964 TT genotype (OR, 0.08; 95% CI, 0.003–1.700; *p *= 0.059, Table 5). Concurrently, a potential risk reduction for cirrhosis was observed with the combination of the rs1800896 AA genotype and the rs1799724 CT/TT genotype (OR, 0.12; 95% CI, 0.007–2.207; *p *= 0.098, Table 5).

**Table 5:** Effects of interaction between rs1800896 and rs1799964/rs1799724 in patients with hepatitis B cirrhosis.

		OR (95% CI)	*p* Value
**rs1800896 AA**	**rs1799964 TT**		
−	−	1.00	0.059
−	+	0.197 (0.004, 10.201)	
+	+	0.076 (0.003, 1.700)	
+	−	0.011 (0.001, 0.331)	
**rs1800896 AA**	**rs1799724 CT/TT**		
−	−	1.00	0.098
−	+	0.001 (0.001, 999.999)	
+	+	0.120 (0.007, 2.207)	
+	−	0.019 (0.001, 0.475)	

Abbreviations: A, adenine; C, cytosine; CI, confidence interval; OR, odds ratio; T, thymine.

## 4 DISCUSSION

Cytokines are key mediators in regulating the inflammatory immune response of the body and can be categorized as pro‐inflammatory or anti‐inflammatory based on their effects, whereas human SNPs can affect the expression or function of cytokines. It has been demonstrated that SNPs within coding regions have the potential to influence protein structure and function by modifying amino acids. Additionally, SNPs situated in noncoding regions that are situated in gene regulatory regions have the capacity to regulate gene expression by affecting transcription factors, which in turn alters the genetic mechanism and affects the pathophysiological process of diseases.^18^ We observed GG/AG dominant model and G allele of rs1800896 were associated with an increased risk of cirrhosis in CHB patients. Instead, rs2227306 CT presented a reduced cirrhosis risk. Rs2055979 AA/AC was negatively associated with the risk of cirrhosis, potentially reversed in males. Rs1799964 CC/CT was positively related to the risk of cirrhosis but reduced the risk of cirrhosis in males. Both rs1799964 TT and rs1799724 CT/TT genotype showed a synergistic effect in reducing the risk of cirrhosis with rs1800896 AA.

Rs1800896 (IL‐10‐1082), one of the most important anti‐inflammatory and immunosuppressive factors regulating the antiviral immune response, is located at a common position in the proximal promoter region of the IL‐10 gene and can affect the transcription quality and quantity of IL‐10.^19^ Our findings observed that CHB patients with the genotype GG/AG (OR, 16.38; 95% CI, 1.13–236.70) and allele G (OR, 5.93; 95% CI, 0.98–36.01) of IL‐10‐1082 were potentially at a higher risk of cirrhosis after adjusting for age, gender, antiviral treatment, HBeAg status, HBV RNA, HBcrAg, and alcohol drinking. Similar results were found by Liu et al. who discovered that allele G was potentially linked to an increased risk of liver cirrhosis in the Chinese population (OR, 2.14; 95% CI, 0.97–1.68).^20^ However, other studies have reported the opposite results. For instance, Yao and colleagues found that the IL‐10‐1082 GA/AA genotype increased the risk of cirrhosis compared to the GG genotype (OR, 1.95; 95% CI, 1.01–3.59).^12^ Similarly, Gao and colleagues reported a significantly higher risk of cirrhosis in HBV‐HCV patients with the AA genotype (OR, 4.26; 95% CI, 1.59–11.36).^21^

There is another IL‐10 locus in our study, rs1800872, specifically located at IL‐10‐592. A meta‐analysis suggested that the IL‐10‐592 heterozygote model was an independent risk factor for LC development in Asian populations (OR, 1.40; 95% CI, 1.03–1.88).^22^ Jin and colleagues discovered that the CC genotype heightened liver cirrhosis risk and was associated with chronic hepatitis B infection in the Chinese population (OR, 2.46; 95% CI, 1.35–4.42).^23^ Moreover, Guo and colleagues found that the IL‐10 GCC haplotype (rs1800896, rs1800871, rs1800872) likely reduced the risk of cirrhosis in HBV‐HCV patients, despite no distinct association observed for these three SNPs in either the general or Asian population.^24^ Magda Rybicka and colleagues also noted that the GCCT haplotype (−1082G/−819C/−592C/−1353T) increased the risk of developing cirrhosis (OR, 2.61; 95% CI, 1.58–4.30).^25^ However, our study did not observe a correlation between IL‐10‐592 and the progression of cirrhosis in patients with chronic HBV infection or any synergistic effect between IL‐10‐592 and IL‐10‐1082. The discrepancies in the above‐mentioned results may be due to varying ethnicities, study designs, disease statuses, and sample sizes. What's more, genetic variant frequencies can also differ across populations from different regions, so further large‐scale studies are needed to confirm our findings.

Tumor necrosis factor (TNF) is a major pro‐inflammatory cytokine that not only promotes immune‐mediated virological control but also causes hepatocellular injury, cirrhosis, and ultimately HCC during the development of liver disease.^26^ TNF‐α is produced by mononuclear macrophages. We examined two SNPs rs1799964 (TNF‐α−1031) and rs1799724 (TNF‐α−857) located in TNF‐α, in addition to three loci, rs1800630 (TNF‐α−863), rs1800629 (TNF‐α−308), and rs361525 (TNF‐α−238), which are located in the promoter regions of the genes and have been reported to influence susceptibility to HBV infection and chronic outcomes such as cirrhosis or HCC.^27^ Previous studies linked the C allele with a heightened HBV infection risk.^8^, ^28^ Conversely, data from Northern Poland showed a higher proportion of the TT genotype among CHB patients.^29^ However, an association between TNF‐α−1031 T/C and cirrhosis risk was not observed in our study. Notably, potential interaction was found when we built an interaction item for gender and TNF‐α−1031. The CC/CT genotype was positively associated with the risk of cirrhosis, but this association could be reversed in male participants. Meanwhile, TNF‐α−857 showed no association with the risk of cirrhosis in our study, aligning with a study by Panigrahi et al.^30^ However, it has been shown that the TNF‐α−857 T allele was an independent risk factor for the development of HCC in patients with HBV‐associated LC (HR, 6.29; 95% CI, 1.62–24.43).^31^ A meta‐analysis also showed a significant association between TNF‐α−857 C/T and HCC risk (OR, 1.31; 95% CI, 1.06–1.62).^27^ These discrepancies may stem from limited sample sizes and differing genetic distribution frequencies across various genders or populations. Inconsistent results may arise due to small sample sizes and different genetic variation frequencies across various populations. Thus, comprehensive longitudinal studies with a larger scale are needed to further explore TNF‐α gene polymorphism in different regional populations, considering all other cirrhosis‐related risk factors to validate these observations.

Rs2227306 (IL‐8‐781), a member of the CXC chemokine superfamily, can elicit a wide range of pro‐inflammatory responses. Previous studies on the effect of IL‐8‐781 gene polymorphism on HBV‐associated cirrhosis are limited. It was shown that IL‐8‐781 TT gene polymorphism significantly reduced the risk of HCC (OR, 0.72; 95% CI, 0.57–0.92).^32^ However, our study showed that IL‐8‐781 CT reduced the risk of cirrhosis (OR, 0.22; 95% CI, 0.04–1.38). When stratifying participants by IL‐8‐781 genotype, TT/CT genotype patients showed lower HBV DNA levels, which may also be the reason for the lower risk of LC.

Previous studies have evaluated the interactions between the pro‐inflammatory cytokines IL‐2‐330 (rs2069762), IFN‐γ+874 (rs2430561), and the anti‐inflammatory cytokines IL‐10‐592 (rs1800872), IL‐10‐1082 (rs1800896).^7^ Therefore, we explored the effect of the pro‐inflammatory properties of IL‐2‐330 (rs2069762), IL‐8 (rs2227306), TNF‐α−857 (rs1799724), and TNF‐α−1031 (rs1799964) versus the anti‐inflammatory properties of IL‐10‐1082 (rs1800896), IL‐10‐592 (rs1800872) on the risk of cirrhosis. Our study demonstrated that rs1800896 AA and rs1799964 TT showed an interaction on reducing the risk of cirrhosis and a similar effect with rs1800896 AA and rs1799724 CT/TT genotype, suggesting IL‐10 and TNF‐α may have a synergistic effect in LC risk reduction. IL‐10 can exert its anti‐inflammatory effects by inhibiting the release of inflammatory factors from T cells, NK cells, and monocyte macrophages. This attenuates the extent of the hepatic inflammatory response.^33^ Concurrently, research has demonstrated that IL‐10 can diminish the synthesis and secretion of TNF‐α by inhibiting the activation of the NF‐κB pathway. The NF‐κB signaling pathway represents a pivotal pathway for TNF‐α‐induced inflammatory responses, thereby regulating hepatocyte apoptosis and necrosis.^34^, ^35^

Furthermore, we investigated the influence of the interaction between gender and SNPs on the development of cirrhosis. Our study observed a potential interaction between gender and rs2055979, and rs1799964, which need more epidemiology and experimental studies to illustrate. It has been demonstrated that in women, the rs1799964 TT genotype in the high dietary inflammatory index group is associated with an elevated risk of gastric cancer (OR, 2.30; 95% CI, 1.27–4.24).^36^ Furthermore, evidence indicates that the TC+CC genotype at the rs5275 locus of the COX‐2 gene is associated with a reduced risk of HCC in women (OR, 1.56; 95% CI, 1.03–2.37).^37^ However, previous studies have demonstrated no gender‐specific associations between rs2055979 and cancer risk. Consequently, research on the correlation between gender and SNPs in cirrhosis remains limited and requires further investigation through a substantial number of studies.

Before offering any recommendations, it's essential to acknowledge the study's limitations. Firstly, our analysis was regionally confined to Wuwei City, and the study subjects were hospital‐based populations, leading to potential selection bias and geographical limitations. Secondly, we didn't consider the likelihood of gene‐environment interactions or linkage disequilibrium between gene polymorphisms. Lastly, our study's relatively small sample size might have limited the statistical power to identify group differences. Therefore, larger‐scale studies in various ethnic groups are necessary to corroborate our findings.

## 5 CONCLUSION

In summary, this study investigated the association between SNPs and the risk of HBV‐related LC. Findings suggested a potential association between the risk of cirrhosis with rs1800896 and rs2227306 polymorphisms. For the first time, the study highlights that the rs2055979 AA/AC and rs1799964 CC/CT polymorphism interact with gender and its potential reversal of cirrhosis risk in males. Notably, we focused on the combined effects of genetic polymorphisms, demonstrating that both rs1799964 TT and rs1799724 CT/TT genotypes showed a synergistic effect in reducing the risk of cirrhosis with rs1800896 AA, potentially inhibiting the progression of HBV infection to cirrhosis.

## AUTHOR CONTRIBUTIONS

Weilu Zhang, Xiaojie Yuan, and Zhongjun Shao designed the study and revised the manuscript. Yijun Li analyzed the data. Haowei Zhou and Xiaojie Yuan did an SNP laboratory examination. Yijun Li and Xiaojie Yuan wrote the manuscript. Weikang Wu collected blood samples and did an HBV‐related biomarkers examination. Wenhua Zhang and Yancheng Ye managed the cohort. Wenling Jia, Chunhui Liang, Haitao Tang, and Fengmei Wang collected data. All authors read and approved the final manuscript.

## CONFLICT OF INTEREST STATEMENT

The authors declare no conflict of interest.

## ETHICS STATEMENT

The studies involving human participants were reviewed and approved by The Clinical Trial Ethics Committee of Gansu Wuwei Medical Academy Cancer Hospital (2024‐Ethics Approval‐10). The patients/participants provided written informed consent to participate in this study.

## Supporting information

Supporting information.

## Data Availability

Original contributions generated for this study are included in the article/Supporting Information. Further inquiries can be directed to the corresponding authors.
