# Supplementary material for: Associations between single nucleotide polymorphisms of cytokines and hepatitis B virus‐related liver cirrhosis: A case‐control study
Source: Immun Inflamm Dis. 2024 Sep 24;12(9):e70017. doi: 10.1002/iid3.70017 (PMC11421045; doi:10.1002/iid3.70017)
Supplement: Supplementary file 1 — Supporting information. [file IID3-12-e70017-s001.docx]

**SUPPLEMENTAL MATERIALS**

**Figure S1** Work-flow diagram

**Figure S2** Percentage of subtypes of human cytokines-related SNPs between CHB and LC groups

**Table S1** Primers and Probes of single nucleotide polymorphisms related to cytokines

**Table S2** Risk factors associated with hepatitis B cirrhosis

**Table S3** SNP Distribution and Hardy-Weinberg equilibrium

**Table S4** Association between SNPs and cirrhosis in chronic hepatitis B infection participants

**Table S5** Association between cytokines-related SNPs and cirrhosis in chronic hepatitis B infection participants (Dominant model)

**Table S6** Association between cytokines-related SNPs and cirrhosis in chronic hepatitis B infection participants (Recessive model)

**Table S7** Association between cytokines-related SNPs and cirrhosis in chronic hepatitis B infection participants (Allelic frequency)

**Table S8** Clinicopathological characteristics of chronic HBV patients stratified by rs1800896 genotypes

**Table S9** Gender stratification analysis of rs1799964

**Table S10** Linkage Disequilibrium tests between single nucleotide polymorphisms

**Figure S1** Work-flow diagram

**case-control study**

**(n = 348)**

**Analysis**

**Patients with chronic hepatitis B infection**

**(n = 375)**

Includtion:

1. Previous HBsAg seropositive results for at least 6 months;

Excluding:

1. Anti-HCV-positive patients (n = 7);
2. Participants with persistent negative SNP results (n = 2);

**LC**

**(n = 45)**

**CHB**

**(n = 45)**

Matching

gender,±5years


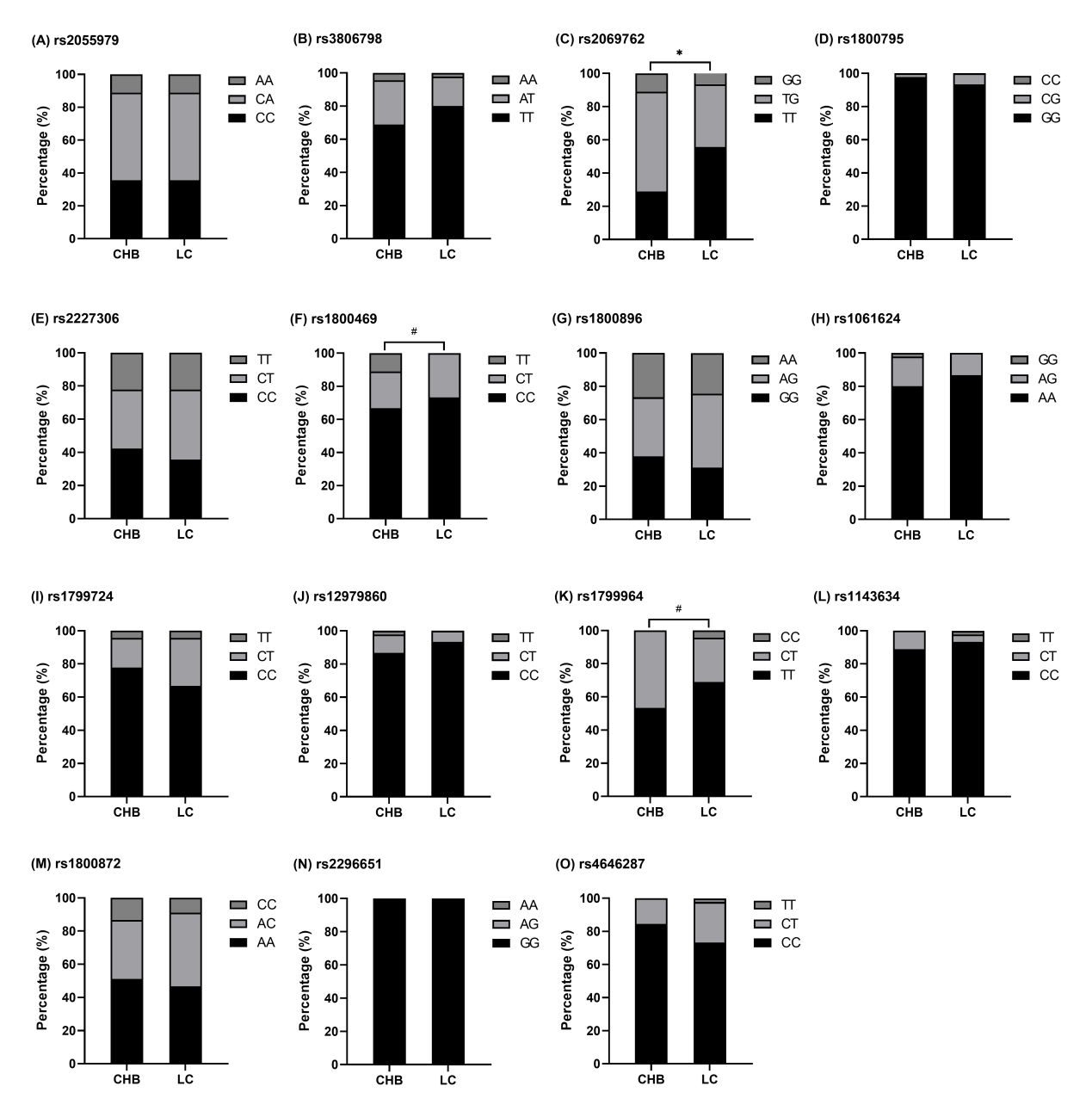


**Figure S2** Percentage of subtypes of human cytokines-related SNPs between CHB and LC groups（^*^*P* < 0.05;^#^*P* < 0.10）

**Table S1** Primers and Probes of single nucleotide polymorphisms related to cytokines

|  | Primer | Probe | Allele |
| --- | --- | --- | --- |
| rs2055979 | |  |  |
| Forward | AGCACATTCAGCTTATTGGAAAGA | ATTTCTAGCATCTCATTCA | T |
| Reverse | GGAAACTCTGGAAAGAACTCTAACCA | ATTTCTAGCATCTCATGCA | G |
| rs3806798^†^ | |  |  |
| Forward | GCACAGGCTGCAAGAAGGA | CTTTGATGTCCAACTAG | T |
| Reverse | GGGTGTCTTCTTCCAACATTTCC | CTTTGAAGTCCAACTAG | A |
| rs2069762 | |  |  |
| Forward | TCCACCACAATATGCTATTCACATG | CAGTGTAGTTTTATGACAAA | T |
| Reverse | CGCCTTCTGTATGAAACAGTTTTTC | TCAGTGTAGTTTTAGGACAA | G |
| rs1800795^†^ | |  |  |
| Forward | GATTGTGCAATGTGACGTCCTT | AGCATGGCAAGACA | G |
| Reverse | GCTGCACTTTTCCCCCTAGTT | AGCATCGCAAGACA | C |
| rs2227306^†^ | |  |  |
| Forward | CCTAGCCCTTGACCTCAGTTAGTT | TGACAACATTGAACGAC | G |
| Reverse | ACCATGAAGATGTTGATATTGTACAAAAAG | AACATTGAACAACTTC | A |
| rs1800469 | |  |  |
| Forward | TGGGAGGTGCTCAGTAAAGGA | CCTTCCATCCTTCA | T |
| Reverse | GGTAGGAGAAGAGGGTCTGTCAAC | CCCTTCCATCCCTCAG | C |
| rs1800896^†^ | |  |  |
| Forward | GGTCCCTTACTTTCCTCTTACCTATCC | TACTTCCCCTTCCCAAA | T |
| Reverse | ACACACAAATCCAAGACAACACTACTAA | TACTTCCCCCTCCC | C |
| rs1061624^†^ | |  |  |
| Forward | CCATGGCAGCAGAGGCTTT | CACAACTCGCTGCC | T |
| Reverse | TTCTGGGCCAAGTTCCTCTAGT | ACAACCCGCTGCC | C |
| rs1799724 | |  |  |
| Forward | GGAGAATGTCCAGGGCTATGG | CCCCCCCTTAATGA | T |
| Reverse | TGGAGGTCCTGGAGGCTCTT | ACCCCCCCTTAACGA | C |
| rs12979860 | |  |  |
| Forward | TGCCTGTCGTGTACTGAACCA | TCCCCGAAGGCGTGA | T |
| Reverse | GAGCGCGGAGTGCAATTC | CGAAGGCGCGAAC | C |
| rs1799964 | |  |  |
| Forward | GGTGAGGCCGCCAGACT | AGAAGATGAAGGAAAAG | T |
| Reverse | TCTCCTGTAACCCATTCCTCAGA | AAGCTGAGAAGACGAA | C |
| rs1143634 | |  |  |
| Forward | GCCTGCCCTTCTGATTTTATACC | TTCAGAACCTATCTTCTTTGA | T |
| Reverse | CGTGCACATAAGCCTCGTTATC | TCAGAACCTATCTTCTTCGA | C |
| rs1800872 | |  |  |
| Forward | TGTGCCTGAGAATCCTAATGAAATC | CCGCCTGTCCTGTAG | C |
| Reverse | AAGCAGCCCTTCCATTTTACTTT | ACCCCGCCTGTACT | A |
| ^†^ Probe was designed in reverse sequence.  A, adenine; T, thymine; C, cytosine; G, guanine. | | | |

| **Table S2** Risk factors associated with hepatitis B cirrhosis | | | | |
| --- | --- | --- | --- | --- |
| Variables | Univariate model | | Multivariate model^§^ | |
|  | OR (95%CI) | *P* | OR (95%CI) | *P* |
| Age,years | 0.98 (0.93-1.04) | 0.539 |  |  |
| Gender | 1.00 (0.42-2.40) | 1.000 |  |  |
| Smoking | 1.72 (0.74-3.99) | 0.205 |  |  |
| Drinking | 0.37 (0.07-2.03) | 0.253 |  |  |
| Antiviral Treatment | 32.24 (6.93-150.10) | **<0.001** | 110.31 (12.32-987.63) | **<0.001** |
| HBeAg, log_10_ IU/mL | 0.23 (0.07-0.69) | **0.009** | 0.17 (0.04-0.80) | **0.025** |
| HBV DNA, log_10_ IU/mL | 0.49 (0.32-0.76) | **0.002** |  |  |
| HBV RNA, log_10_ IU/mL | 0.58 (0.40-0.84) | **0.004** | 0.49 (0.28-0.86) | **0.013** |
| HBcrAg, log_10_ U/mL | 0.91 (0.72-1.14) | 0.399 |  |  |
| Course |  |  |  |  |
| <5 years | 1.00 |  |  |  |
| ≥5 years | 0.96 (0.38-2.42) | 0.274 |  |  |
| Unknown | 0.34 (0.10-1.15) | 0.067 |  |  |
| ALT, IU/L | 0.99 (0.98-1.00) | 0.182 |  |  |
| CAP, dB/m | 0.99 (0.98-1.00) | **0.036** |  |  |

^§^Adjusting for age, gender, smoking, alcohol drinking, antiviral treatment, HBeAg status, HBV RNA, and HBcrAg.

HBeAg, Hepatitis B e antigen; HBV, Hepatitis B virus; HBcrAg, Hepatitis B c antigen; ALT, alanine aminotransferase; CAP, controlled attenuation parameter; OR, odds ratio; CI, confidence interval.

| **Table S3** SNP Distribution and Hardy-Weinberg equilibrium | | | | | | | |
| --- | --- | --- | --- | --- | --- | --- | --- |
| SNP | Cytokines | Allele | AAF globally ^†^ | AAF in Asian^†^ | AAF in this study | *χ*^2^ | *P*^‡^ |
| rs2055979 | IL-21 | C>A | 0.277 | 0.420 | 0.378 | 1.627 | >0.05 |
| rs3806798 | IL-15 | T>A | 0.104 | 0.161 | 0.144 | 0.916 | >0.05 |
| rs2069762 | IL-2 | A>C | 0.289 | 0.329 | 0.333 | 0.900 | >0.05 |
| rs1800795 | IL-6 | C>G | 0.639 | 1.000 | 0.978 | 0.046 | >0.05 |
| rs2227306 | CXCL8/IL-8 | C>T | 0.362 | 0.422 | 0.417 | 3.600 | >0.05 |
| rs1800469 | TGF-β1 | A>G | 0.672 | 0.509 | 0.822 | 2.416 | >0.05 |
| rs1800896 | IL-10/IL-19 | T>C | 0.453 | 0.006 | 0.089 | 0.141 | >0.05 |
| rs1061624 | TNFRSF1B | A>G | 0.541 | 0.467 | 0.544 | 3.374 | >0.05 |
| rs1799724 | TNF-α | C>T | 0.121 | 0.105 | 0.161 | 1.684 | >0.05 |
| rs12979860 | IFNL4/IL-28B | C>T | 0.328 | 0.040 | 0.056 | 2.105 | >0.05 |
| rs1799964 | TNF-α | T>C | 0.211 | 0.210 | 0.206 | 1.354 | >0.05 |
| rs1143634 | IL-1β | G>A | 0.228 | 0.037 | 0.050 | 2.958 | >0.05 |
| rs1800872 | IL-10/IL-19 | T>G | 0.706 | 0.274 | 0.311 | 0.402 | >0.05 |
| rs2296651 | SLC10A1 | G>A | 0.002 | 0.044 | 0.000 | 0.000 | >0.05 |
| rs4646287 | SLC10A1 | C>T | 0.001 | 0.100 | 0.111 | 0.014 | >0.05 |
| ^†^From National Center for Biotechnology Information.  ^‡^df = 1, *χ*^2^ > 3.84, *P* < 0.05; df = 2, *χ*^2^ > 5.99, *P* < 0.05.  HWE, Hardy-Weinberg equilibrium; AAF, alternative allele frequency; IL, interleukin; CXCL, C-X-C motif ligand; TGF-β1, transforming growth factor beta-1; TNFRSF1B, tumor necrosis factor receptor superfamily, member 1B; TNF-α, tumor necrosis factor alpha; IFN, interferon; SLC10A1, solute carrier family 10 member 1. | | | | | | | |

| **Table S4** Association between SNPs and cirrhosis in chronic hepatitis B infection participants | | | | | | |
| --- | --- | --- | --- | --- | --- | --- |
| Genotype | CHB  (n=45) | LC  (n=45) | Univariate model | | Multivariate model^§^ | |
|  |  |  | OR (95% CI) | *P* | OR (95% CI) | *P* |
| **rs2055979** |  |  |  |  |  |  |
| CC | 16 (35.6) | 16 (35.6) | 1.00 |  | 1.00 |  |
| AC | 24 (53.3) | 24 (53.3) | 1.00(0.41-2.45) | 1.000 | 1.46(0.33-6.44) | 0.834 |
| AA | 5 (11.1) | 5 (11.1) | 1.00(0.24-4.14) | 1.000 | 1.47(0.08-25.41) | 0.889 |
| **rs3806798** |  |  |  |  |  |  |
| TT | 31 (68.9) | 36 (80.0) | 1.00 |  | 1.00 |  |
| AT | 12 (26.7) | 8 (17.8) | 0.57(0.21-1.59) | 0.863 | 0.38(0.07-2.07) | 0.306 |
| AA | 2 (4.4) | 1 (2.2) | 0.43(0.04-4.98) | 0.652 | 2.10(0.04-116.40) | 0.553 |
| **rs1800795** |  |  |  |  |  |  |
| GG | 44 (97.8) | 42 (93.3) | 1.00 |  | 1.00 |  |
| CG | 1 (2.2) | 3 (6.7) | 3.14(0.31-31.42) | 0.330 | 2.85(0.01-999.99) | 0.780 |
| CC | 0 (0.0) | 0 (0.0) | - | - | - | - |
| **rs1800469** |  |  |  |  |  |  |
| CC | 30 (66.7) | 33 (73.3) | 1.00 |  | 1.00 |  |
| CT | 10 (22.2) | 12 (26.7) | 1.09(0.41-2.89) | 0.950 | 0.84(0.15-4.85) | 0.971 |
| TT | 5 (11.1) | 0 (0.0) | - | - | - | - |
| **rs1061624** |  |  |  |  |  |  |
| GG | 17 (37.8) | 14 (31.1) | 1.00 |  | 1.00 |  |
| AG | 16 (35.6) | 20 (44.4) | 1.52(0.58-3.99) | 0.402 | 0.76(0.12-4.91) | 0.979 |
| AA | 12 (26.6) | 11 (24.4) | 1.11(0.38-3.28) | 0.834 | 0.61(0.09-3.96) | 0.681 |
| **rs1799724** |  |  |  |  |  |  |
| CC | 35 (77.8) | 30 (66.7) | 1.00 |  | 1.00 |  |
| CT | 8 (17.8) | 13 (28.9) | 1.90(0.69-5.19) | 0.411 | 1.43(0.26-7.91) | 0.482 |
| TT | 2 (4.4) | 2 (4.4) | 1.17(0.16-8.79) | 0.873 | 16.63(0.14-999.99) | 0.288 |
| **rs12979860** |  |  |  |  |  |  |
| CC | 39 (86.7) | 42 (93.3) | 1.00 |  | 1.00 |  |
| CT | 5 (11.1) | 3 (6.7) | 0.56(0.13-2.49) | 0.980 | 0.35(0.04-3.40) | 0.988 |
| TT | 1 (2.2) | 0 (0.0) | - | - | - | - |
| **rs1799964** |  |  |  |  |  |  |
| TT | 24 (53.3) | 31 (68.9) | 1.00 |  | 1.00 |  |
| CT | 21 (46.7) | 12 (26.7) | 0.44(0.18-1.07) | 0.966 | 0.45(0.09-2.21) | 0.968 |
| CC | 0 (0.0) | 2 (4.4) | - | - | - | - |
| **rs1143634** |  |  |  |  |  |  |
| CC | 40 (88.9) | 42 (93.3) | 1.00 |  | 1.00 |  |
| CT | 5 (11.1) | 2 (4.4) | 0.38(0.07-2.08) | 0.975 | 0.30(0.02-3.75) | 0.980 |
| TT | 0 (0.0) | 1 (2.2) | - | - | - | - |
| **rs1800872** |  |  |  |  |  |  |
| AA | 23 (51.1) | 21 (46.7) | 1.00 |  | 1.00 |  |
| AC | 16 (35.6) | 20 (44.4) | 1.37(0.57-3.32) | 0.335 | 3.69(0.69-19.80) | 0.536 |
| CC | 6 (13.3) | 4 (8.9) | 0.73(0.18-2.95) | 0.490 | 4.44(0.28-71.51) | 0.530 |
| **rs2296651** |  |  |  |  |  |  |
| GG | 45 (100.0) | 45 (100.0) | 1.00 |  | 1.00 |  |
| AG | 0 (0.0) | 0 (0.0) | - | - | - | - |
| AA | 0 (0.0) | 0 (0.0) | - | - | - | - |
| **rs4646287** |  |  |  |  |  |  |
| CC | 38 (84.4) | 33 (73.3) | 1.00 |  | 1.00 |  |
| CT | 7 (15.6) | 11 (24.4) | 1.81(0.63-5.20) | 0.970 | 0.82(0.13-5.28) | 0.968 |
| TT | 0 (0.0) | 1 (2.2) | - | - | - | - |
| ^§^Adjusting for age, gender, smoking, alcohol drinking, antiviral treatment, HBeAg status, HBV RNA, and HBcrAg.  A, adenine; T, thymine; C, cytosine; G, guanine; OR, odds ratio; CI, confidence interval. | | | | | | |

| **Table S5** Association between cytokines-related SNPs and cirrhosis in chronic hepatitis B infection participants (Dominant model) | | | | | | |
| --- | --- | --- | --- | --- | --- | --- |
| Genotype | CHB  (n=45) | LC  (n=45) | Univariate model | | Multi-adjusted model^§^ | |
|  |  |  | OR (95% CI) | *P* | OR (95% CI) | *P* |
| **rs2055979** |  |  |  |  |  |  |
| CC | 16 (35.6) | 16 (35.6) | 1.00 |  | 1.00 |  |
| AA/AC | 29 (64.4) | 29 (64.4) | 1.00(0.42-2.37) | 1.000 | 1.46(0.34-6.18) | 0.608 |
| **rs3806798** |  |  |  |  |  |  |
| TT | 31 (68.9) | 36 (80.0) | 1.00 |  | 1.00 |  |
| AA/AT | 14 (31.1) | 9 (20.0) | 0.55(0.21-1.45) | 0.230 | 0.48(0.10-2.39) | 0.371 |
| **rs1800795** |  |  |  |  |  |  |
| GG | 44 (97.8) | 42 (93.3) | 1.00 |  | 1.00 |  |
| CC/CG | 1 (2.2) | 3 (6.7) | 3.14(0.31-31.42) | 0.330 | 2.85(0.01-999.99) | 0.780 |
| **rs1800469** |  |  |  |  |  |  |
| CC | 30 (66.7) | 33 (73.3) | 1.00 |  | 1.00 |  |
| TT/CT | 15 (33.3) | 12 (26.7) | 0.73(0.29-1.80) | 0.491 | 0.67(0.13-3.46) | 0.633 |
| **rs1061624** |  |  |  |  |  |  |
| GG | 17 (37.8) | 14 (31.1) | 1.00 |  | 1.00 |  |
| AA/AG | 28 (62.2) | 31 (68.9) | 1.34(0.56-3.22) | 0.506 | 0.68(0.14-3.24) | 0.627 |
| **rs1799724** |  |  |  |  |  |  |
| CC | 35 (77.8) | 30 (66.7) | 1.00 |  | 1.00 |  |
| TT/CT | 10 (22.2) | 15 (33.3) | 1.75(0.69-4.47) | 0.242 | 2.00(0.41-9.83) | 0.393 |
| **rs12979860** |  |  |  |  |  |  |
| CC | 39 (86.7) | 42 (93.3) | 1.00 |  | 1.00 |  |
| TT/CT | 6 (13.3) | 3 (6.7) | 0.46(0.11-1.99) | 0.301 | 0.35(0.04-3.27) | 0.356 |
| **rs1799964** |  |  |  |  |  |  |
| TT | 24 (53.3) | 31 (68.9) | 1.00 |  | 1.00 |  |
| CC/CT | 21 (46.7) | 14 (31.1) | 0.52(0.22-1.22) | 0.132 | 0.54(0.11-2.60) | 0.442 |
| **rs1143634** |  |  |  |  |  |  |
| CC | 40 (88.9) | 42 (93.3) | 1.00 |  | 1.00 |  |
| TT/CT | 5 (11.1) | 3 (6.7) | 0.57(0.13-2.55) | 0.463 | 0.33(0.03-3.85) | 0.374 |
| **rs1800872** |  |  |  |  |  |  |
| AA | 23 (51.1) | 21 (46.7) | 1.00 |  | 1.00 |  |
| CC/AC | 22 (48.9) | 24 (53.3) | 1.20(0.52-2.73) | 0.674 | 3.81(0.76-19.17) | 0.104 |
| **rs2296651** |  |  |  |  |  |  |
| GG | 45 (100.0) | 45 (100.0) | 1.00 |  | 1.00 |  |
| AA/AG | 0 (0.0) | 0 (0.0) | - | - | - | - |
| **rs4646287** |  |  |  |  |  |  |
| CC | 38 (84.4) | 33 (73.3) | 1.00 |  | 1.00 |  |
| TT/CT | 7 (15.6) | 12 (26.7) | 1.97(0.70-5.60) | 0.201 | 0.99(0.16-6.16) | 0.991 |
| ^§^Adjusting for age, gender, smoking, alcohol drinking, antiviral treatment, HBeAg status, HBV RNA, and HBcrAg.  A, adenine; T, thymine; C, cytosine; G, guanine; OR, odds ratio; CI, confidence interval. | | | | | | |

| **Table S6** Association between cytokines-related SNPs and cirrhosis in chronic hepatitis B infection participants (Recessive model) | | | | | | |
| --- | --- | --- | --- | --- | --- | --- |
| Genotype | CHB  (n=45) | LC (n=45) | Univariate model | | Multi-adjusted model^§^ | |
|  |  |  | OR (95% CI) | *P* | OR (95% CI) | *P* |
| **rs2055979** |  |  |  |  |  |  |
| CC/AC | 40 (88.9) | 40 (88.9) | 1.00 |  | 1.00 |  |
| AA | 5 (11.1) | 5 (11.1) | 1.00(0.27-3.72) | 1.000 | 1.19(0.08-17.78) | 0.901 |
| **rs3806798** |  |  |  |  |  |  |
| TT/AT | 43 (95.6) | 44 (97.8) | 1.00 |  | 1.00 |  |
| AA | 2 (4.4) | 1 (2.2) | 0.49(0.04-5.59) | 0.565 | 2.40(0.05-112.30) | 0.656 |
| **rs1800795** |  |  |  |  |  |  |
| GG/CG | 45 (100.0) | 45 (100.0) | 1.00 |  | 1.00 |  |
| CC | 0 (0.0) | 0 (0.0) | - | - | - | - |
| **rs1800469** |  |  |  |  |  |  |
| CC/CT | 40 (88.9) | 45 (100.0) | 1.00 |  | 1.00 |  |
| TT | 5 (11.1) | 0 (0.0) | - | - | - | - |
| **rs1061624** |  |  |  |  |  |  |
| GG/AG | 33 (73.3) | 34 (75.6) | 1.00 |  | 1.00 |  |
| AA | 12 (26.7) | 11 (24.4) | 0.89(0.35-2.30) | 0.809 | 0.67(0.12-3.74) | 0.651 |
| **rs1799724** |  |  |  |  |  |  |
| CC/CT | 43 (95.6) | 43 (95.6) | 1.00 |  | 1.00 |  |
| TT | 2 (4.4) | 2 (4.4) | 1.00(0.14-7.43) | 1.000 | 16.46(0.14-999.99) | 0.249 |
| **rs12979860** |  |  |  |  |  |  |
| CC/CT | 44 (97.8) | 45 (100.0) | 1.00 |  | 1.00 |  |
| TT | 1 (2.2) | 0 (0.0) | - | - | - | - |
| **rs1799964** |  |  |  |  |  |  |
| TT/CT | 45 (100.0) | 43 (95.6) | 1.00 |  | 1.00 |  |
| CC | 0 (0.0) | 2 (4.4) | - | - | - | - |
| **rs1143634** |  |  |  |  |  |  |
| CC/CT | 45 (100.0) | 44 (97.8) | 1.00 |  | 1.00 |  |
| TT | 0 (0.0) | 1 (2.2) | - | - | - | - |
| **rs1800872** |  |  |  |  |  |  |
| AA/AC | 39 (86.7) | 41 (91.1) | 1.00 |  | 1.00 |  |
| CC | 6 (13.3) | 4 (8.9) | 0.63(0.17-2.42) | 0.505 | 2.22(0.19-25.95) | 0.527 |
| **rs2296651** |  |  |  |  |  |  |
| GG/AG | 45 (100.0) | 45 (100.0) | 1.00 |  | 1.00 |  |
| AA | 0 (0.0) | 0 (0.0) | - | - | - | - |
| **rs4646287** |  |  |  |  |  |  |
| CC/CT | 45 (100.0) | 44 (97.8) | 1.00 |  | 1.00 |  |
| TT | 0 (0.0) | 1 (2.2) | - | - | - | - |
| ^§^Adjusting for age, gender, smoking, alcohol drinking, antiviral treatment, HBeAg status, HBV RNA, and HBcrAg.  A, adenine; T, thymine; C, cytosine; G, guanine; OR, odds ratio; CI, confidence interval. | | | | | | |

| **Table S7** Association between cytokines-related SNPs and cirrhosis in chronic hepatitis B infection participants (Allelic frequency) | | | | | | |
| --- | --- | --- | --- | --- | --- | --- |
| Genotype | CHB  (n=45) | LC (n=45) | Univariate model | | Multi-adjusted model^§^ | |
|  |  |  | OR (95% CI) | *P* | OR (95% CI) | *P* |
| **rs2055979** |  |  |  |  |  |  |
| C | 56 (62.2) | 56 (62.2) | 1.00 |  | 1.00 |  |
| A | 34 (37.8) | 34 (37.8) | 1.00(0.55-1.83) | 1.000 | 1.25(0.44-3.57) | 0.676 |
| **rs3806798** |  |  |  |  |  |  |
| T | 74 (82.2) | 80 (88.9) | 1.00 |  | 1.00 |  |
| A | 16 (17.8) | 10 (11.1) | 0.58(0.25-1.35) | 0.207 | 0.67(0.17-2.57) | 0.555 |
| **rs1800795** |  |  |  |  |  |  |
| G | 89 (98.9) | 87 (96.7) | 1.00 |  | 1.00 |  |
| C | 1 (1.1) | 3 (3.3) | 3.07(0.31-30.08) | 0.336 | 2.81(0.01-999.99) | 0.780 |
| **rs1800469** |  |  |  |  |  |  |
| C | 70 (77.8) | 78 (86.7) | 1.00 |  | 1.00 |  |
| T | 20 (22.2) | 12 (13.3) | 0.54(0.25-1.18) | 0.122 | 0.61(0.15-2.43) | 0.482 |
| **rs1061624** |  |  |  |  |  |  |
| G | 50 (55.6) | 48 (53.3) | 1.00 |  | 1.00 |  |
| A | 40 (44.4) | 42 (46.7) | 1.09(0.61-1.97) | 0.765 | 0.74(0.27-2.05) | 0.557 |
| **rs1799724** |  |  |  |  |  |  |
| C | 78 (86.7) | 73 (81.1) | 1.00 |  | 1.00 |  |
| T | 12 (13.3) | 17 (18.9) | 1.51(0.68-3.39) | 0.313 | 2.27(0.55-9.29) | 0.256 |
| **rs12979860** |  |  |  |  |  |  |
| C | 83 (92.2) | 87 (96.7) | 1.00 |  | 1.00 |  |
| T | 7 (7.8) | 3 (3.3) | 0.41(0.10-1.63) | 0.206 | 0.38(0.05-3.11) | 0.364 |
| **rs1799964** |  |  |  |  |  |  |
| T | 69 (76.7) | 74 (82.2) | 1.00 |  | 1.00 |  |
| C | 21 (23.3) | 16 (17.8) | 0.71(0.34-1.47) | 0.358 | 0.82(0.25-2.72) | 0.744 |
| **rs1143634** |  |  |  |  |  |  |
| C | 85 (94.4) | 86 (95.6) | 1.00 |  | 1.00 |  |
| T | 5 (5.6) | 4 (4.4) | 0.79(0.21-3.05) | 0.733 | 0.41(0.04-3.87) | 0.436 |
| **rs1800872** |  |  |  |  |  |  |
| A | 62 (68.9) | 62 (68.9) | 1.00 |  | 1.00 |  |
| C | 28 (31.1) | 28 (31.1) | 1.00(0.53-1.88) | 1.000 | 2.53(0.78-8.19) | 0.122 |
| **rs2296651** |  |  |  |  |  |  |
| G | 90 (100.0) | 90 (100.0) | 1.00 |  | 1.00 |  |
| A | 0 (0.0) | 0 (0.0) | - | - | - | - |
| **rs4646287** |  |  |  |  |  |  |
| C | 83 (92.2) | 77 (85.6) | 1.00 |  | 1.00 |  |
| T | 7 (7.8) | 13 (14.4) | 2.00(0.76-5.28) | 0.161 | 1.22(0.26-5.80) | 0.801 |
| ^§^Adjusting for age, gender, smoking, alcohol drinking, antiviral treatment, HBeAg status, HBV RNA, and HBcrAg.  A, adenine; T, thymine; C, cytosine; G, guanine; OR, odds ratio; CI, confidence interval. | | | | | | |

| **Table S8** Clinicopathological characteristics of chronic HBV patients stratified by rs2227306 genotypes | | | |
| --- | --- | --- | --- |
| Characteristics | CC  (n=35) | TT/CT  (n=55) | *P* |
| Age,years | 55.5 (8.1) | 54.5 (7.6) | 0.589 |
| Gender,male (%) | 23.0 (65.7) | 37.0 (67.3) | 0.879 |
| Smoking, n (%) | 16.0 (45.7) | 24.0 (43.6) | 0.847 |
| Drinking, n (%) | 2.0 (5.7) | 5.0 (9.1) | 0.552 |
| Treatment, n (%) | 21.0 (60.0) | 40.0 (72.7) | 0.208 |
| HBeAg(+), n (%) | 8.0 (22.9) | 13.0 (23.6) | 0.932 |
| HBV DNA, log_10_ IU/mL | 2.4 (2.0-4.2) | 2.0 (2.0-3.1) | **0.034** |
| HBV RNA, log_10_ IU/mL | 2.3 (2.0-3.5) | 2.2 (2.0-3.7) | 0.686 |
| HBcrAg, log_10_ U/mL | 4.7 (3.2-5.8) | 4.6 (3.1-5.8) | 0.744 |
| ALT, IU/L | 41.1 (23.8-64.0) | 37.3 (25.9-61.3) | 0.974 |
| AST, IU/L | 37.3 (25.3-49.8) | 31.3 (24.4-50.6) | 0.593 |
| CAP, dB/m | 222.0 (193.0-254.0) | 223.0 (189.0-256.0) | 0.895 |
| LSM, kPa | 7.8 (6.1-13.9) | 8.1 (4.7-16.3) | 0.679 |
| HBeAg, Hepatitis B e antigen; HBV, Hepatitis B virus; HBcrAg, Hepatitis B c antigen; ALT, alanine aminotransferase; AST, aspartate aminotransferase; CAP, controlled attenuation parameter; LSM, liver stiffness measurement; A, adenine; T, thymine; C, cytosine; G, guanine. | | | |

| **Table S9** Gender stratification analysis of rs1799964 | | | | | |
| --- | --- | --- | --- | --- | --- |
| rs1799964 CC/CT | *β* | SE | Wald X^2^ | *P* | OR (95%CI) |
| gender |  |  |  |  |  |
| Male | -1.008 | 0.460 | 4.805 | 0.028 | 0.133 (0.022-0.808) |
| Female | 7.430 | 94.850 | 0.006 | 0.938 | 999.999 (0.001-999.999) |

| **Table S10** Linkage Disequilibrium tests between single nucleotide polymorphisms | | | | | |
| --- | --- | --- | --- | --- | --- |
| r^2^ | rs2227306 | rs1799724 | rs1799964 | rs1800896 | rs1800872 |
| rs2069762 | 0.001 | 0.000 | 0.023 | 0.001 | 0.000 |
| rs2227306 | - | 0.000 | 0.023 | 0.009 | 0.000 |
| rs1799724 | - | - | 0.017 | 0.019 | 0.025 |
| rs1799964 | - | - | - | 0.032 | 0.027 |
| rs1800896 | - | - | - | - | 0.216 |
| r^2^, correlation coefficient. | | | | | |
